# Alternative Routes of Zoonotic Vaccinia Virus Transmission, Brazil

**DOI:** 10.3201/eid2112.141249

**Published:** 2015-12

**Authors:** Galileu B. Costa, Iara A. Borges, Pedro A. Alves, Júlia B. Miranda, Ana Paula M.F. Luiz, Paulo C.P. Ferreira, Jônatas S. Abrahão, Elizabeth C. Moreno, Erna G. Kroon, Giliane de Souza Trindade

**Affiliations:** Universidade Federal de Minas Gerais, Belo Horizonte, Minas Gerais, Brazil (G.B. Costa, I.A. Borges, P.A. Alves, J.B. Miranda, A.P.M.F. Luiz, P.C.P. Ferreira, J.S. Abrahão, E.G. Kroon, G. de Souza Trindade);; Fundação Hemominas. Belo Horizonte (E.C. Moreno)

**Keywords:** vaccinia, vaccinia virus, VACV, viruses, transmission, alternative routes, cows, horses, zoonoses, Brazil

**To the Editor:** Vaccinia virus (VACV) causes exanthematous disease (bovine vaccinia) in Brazil. Outbreaks of this disease in humans have been reported since the late 1990s and have spread throughout Brazil (*1*). Natural human infections with VACV occur by close contact with infected cattle during milking. Lesions can spread to secondary body sites (forearms, arms, and face). Thus, person-to-person transmission occurs (*1*).

Moreover, virus can persist in household environments, remain infectious, and be transmitted by fomites (*2*). Although raw milk and cheese are potential sources of infection, no clinical cases have been associated with this transmission route (*3**,**4*). Data for person-to-person transmission in Brazil are scarce, but person-to-person transmission was recently reported (*5*). We report a possible case of person-to-person transmission of VACV.

This study was approved by the Research Ethics Committee of Universidade Federal de Minas Gerais (registration protocol FR-413704). In September 2012, during a serologic survey in a rural area of Serro City (18°36′17″S, 43°22′46″W), Minas Gerais, Brazil (Technical Appendix Figure, panel A), blood samples were obtained from a family of 5 persons (father, mother, and 3 daughters). The father and mother were 48 and 53 years of age, respectively, and had been vaccinated against smallpox. They reported contact with cows and horses (Technical Appendix Table 1). Only the father had milked cows. The 3 daughters (13, 13, and 14 years of age) did not engage in any exposure activity. However, all family members had consumed raw milk and cheese.

Bovine vaccinia lesions were observed on the hand of the father (Technical Appendix Figure, panel B). In 2011, he had vesicular disease (no laboratory diagnosis) with clinical and epidemiologic features (lesions) suggestive of bovine vaccinia on his hands and forearms and systemic symptoms (fever, headache, malaise, myalgia, lymphadenopathy, and abdominal pain). His symptoms were mild and without any systemic clinical features. Two lesions developed on his hands and dried swab samples were collected from both lesions. Swab samples were processed as described (*2*) and used for virus isolation and molecular diagnosis.

On the basis of previous studies that detected viral DNA in clinical samples from persons with bovine vaccinia (*1*), we used a quantitative PCR to amplify the *vgf* and *ha* genes of VACV (*3**–**5*), a standard PCR to detect the *ha* gene (*3**–**5*), and a seminested PCR to detect the *ati* gene (F.L. Assis, unpub. data). Serum samples were used for detection of virus-neutralizing antibodies (orthopoxvirus 50% plaque-reduction neutralization test) and molecular diagnostic studies (*1*). Virus isolation was attempted in Vero cells and chorioallantoic membrane. All results were negative.

The 50% plaque-reduction neutralization test showed that the father, mother, and 14-year-old daughter had neutralizing antibodies against orthopoxvirus (titers 800, 3,200, and 800 neutralizing units/mL, respectively). All family members had positive results by molecular diagnostic test for >1 virus gene (Technical Appendix Table 1). To rule out infection with parapopoxvirus, a complementary PCR (*6*) was also performed, and all family members had negative results.

Quantitative PCR products for the *ha* gene from 3 virus-positive samples were sequenced in both directions in triplicate (Mega BACE Sequencer; GE Healthcare, Little Chalfont, UK). Sequences were aligned by using ClustalW (http://www.genome.jp/tools/clustalw/) and MEGA4.1 (http://www.megasoftware.net/) and showed 100% identity with each other (Figure). A phylogenetic tree was constructed by using the neighbor-joining method and 1,000-bootstrap replicates in the Tamura-3 parameter model (MEGA4.1). Sequences were grouped with VACV group 2 isolates. Sequences obtained were deposited in GenBank under accession nos. KP889223–5).

**Figure:**
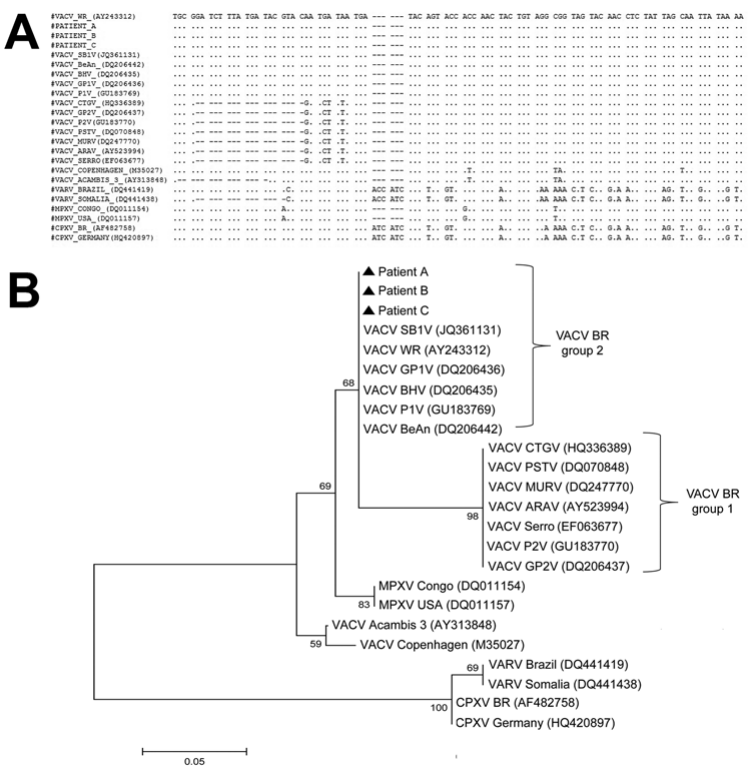
A) Nucleotide sequence of vaccinia virus (VACV) hemagglutinin gene and homologous sequences of several orthopoxviruses, Brazil. Dots indicate sequence identity; dashes indicate deletions. VARV, variola virus; MPXV, monkeypox virus; CPXV, cowpox virus. B) Consensus phylogenetic tree based on nucleotide sequences of orthopoxvirus hemagglutinin genes. Tree was constructed with hemagglutinin gene sequences by using the neighbor-joining method with 1,000 bootstrap replicates and the Tamura 3-parameter model in MEGA4 (http://www.megasoftware.net/). Strains had the deletion region conserved and were grouped with other VACV (group 2) isolated in Brazil. Numbers along branches are bootstrap values. Scale bar indicates nucleotide substitutions per site.

In Brazil, outbreaks of bovine vaccinia are associated with rural environments. However, some clinical and epidemiologic aspects remain unclear. The infection of the father was associated with direct contact with cattle. Immunity conferred by smallpox vaccination did not prevent infection; this lack of immune response has been demonstrated in other studies in Brazil (*7*). Long-term protection might require multiple virus exposures, and severity of poxvirus infections might be influenced by the immunologic state of the host and virulence of virus strains (*1*,*8**,**9*).

The mother and 2 daughters with virus DNA in blood samples and the 14-year-old daughter with high titers of virus-neutralizing antibodies suggest that alternative routes (other than milking) for VACV infection of humans should be considered. These alternative routes can include person-to-person or environmental transmission because the 2 daughters did not report any exposure activities related to milking or contact with cattle (Technical Appendix Tables 1, 2). Persistence of VACV in household environments has been reported (*2**,**10*). The family also consumed raw milk and cheese, a common practice in the region. Therefore, infection with VACV through raw milk and cheese consumption should also be considered. The patients did not report oral lesions or a history of skin/mucosal lesions.

In conclusion, person-to-person transmission of VACV in these cases might have been caused by direct contact between the father and family members, contact with virus in the home, or consumption of unpasteurized milk and cheese. Additional studies are necessary to elucidate the role of these transmission pathways in spread of VACV in Brazil.

**Technical Appendix.** Additional information on alternative routes of zoonotic vaccinia virus transmission, Brazil.
