# Supplementary material for: Alternative Routes of Zoonotic Vaccinia Virus Transmission, Brazil
Source: Emerg Infect Dis. 2015 Dec;21(12):2244–6. doi: 10.3201/eid2112.141249 (PMC4672421; doi:10.3201/eid2112.141249)
Supplement: Supplementary file 1 — Technical Appendix. Additional information on alternative routes of zoonotic vaccinia virus transmission, Brazil. [file 14-1249-Techapp-s1.pdf]

Article DOI: http://dx.doi.org/10.3201/eid2112.141249

Alternative Routes of Zoonotic Vaccinia Virus Transmission, Brazil

Technical Appendix

Technical Appendix Table 1. Epidemiologic data for 5 family members regarding exposure to vaccinia virus, Brazil

	Patient				
Characteristic	Father	Mother	Twin daughter	Twin daughter	Daughter
Occupation	Rural worker	Rural worker	Student	Student	Student
Contact with cows and horses	Yes	Yes	No	No	No
Contact with the environment	No	No	No	No	No
Vaccinated	Yes	Yes	No	No	No
Consumption of raw milk/cheese	Yes	Yes	Yes	Yes	Yes
Milked cows	Yes	No	No	No	No
Cheese production	Yes	Yes	No	No	No

Technical Appendix Table 2. Diagnostic results for 5 patients exposed to vaccinia virus, Brazil*

		Serologic result	Quantitative PCR result		Standard PCR result		
Patient	Virus isolation	(neutralizing units/mL)	*vgf*	*ha*	*vgf*	*ati*†	*ha*
Father	–	+ (800)	+	+	+	+	–
Mother	ND	+ (3,200)	+	+	–	-	–
Twin daughter	ND	–	–	+	–	+	–
Twin daughter	ND	–	+	–	–	-	–
Daughter	ND	+ (800)	–	–	–	+	–

*PCR was performed with serum and swab samples from patient A. Other PCRs were performed only with serum samples. *vgf*, virus growth factor gene; *ha*, hemagglutinin gene; *ati*, A-type inclusion gene; –, negative; +, positive; ND, not done.
†By seminested PCR.

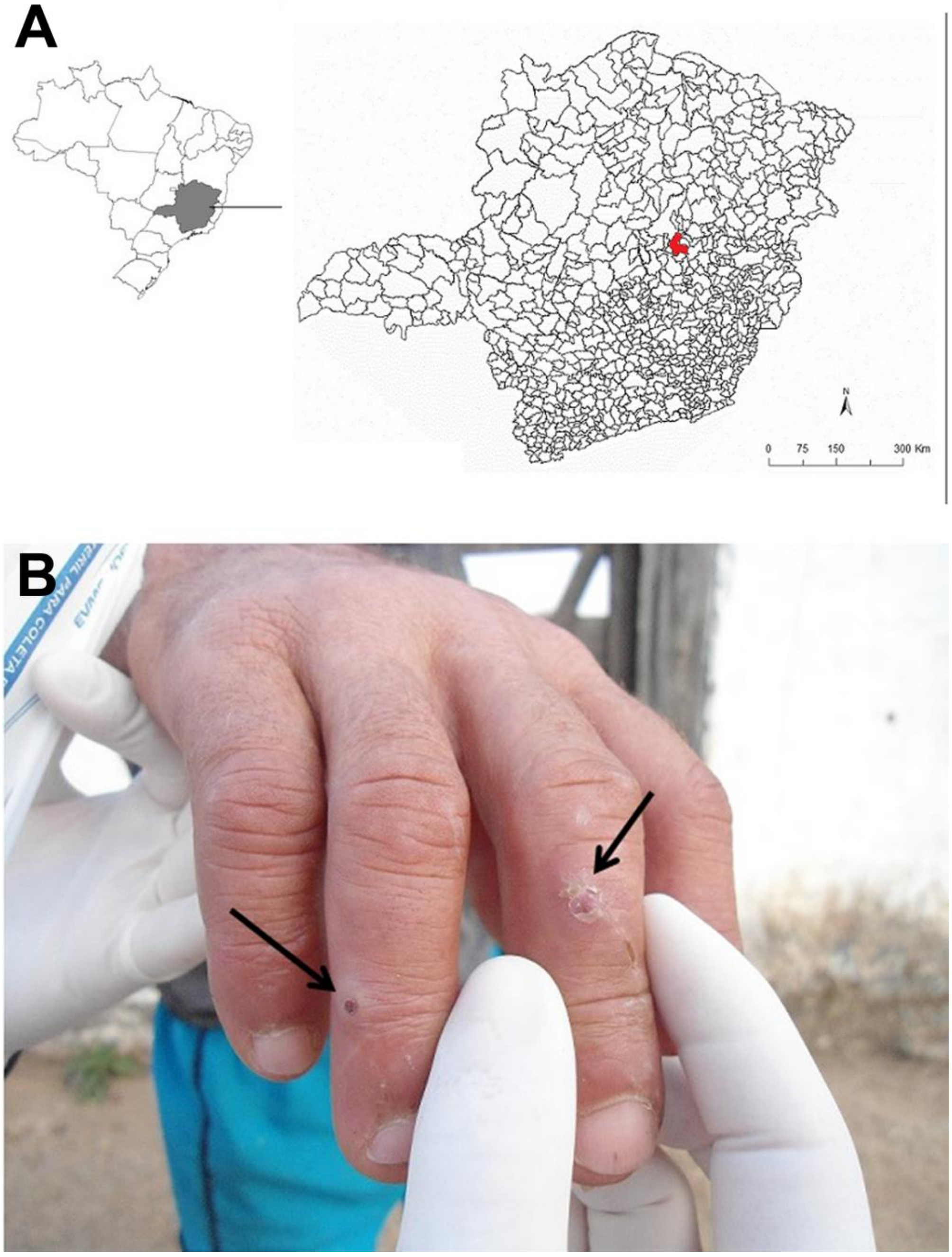

Technical Appendix Figure. A) Minais Gerais, Brazil (gray area) and Serro City (red area). B) Lesions (arrows) on the hands of a family member (father). These lesions were similar to those observed during outbreaks of bovine vaccinia, Brazil.
